# Diltiazem-Induced Reversible Cardiogenic Shock in Thyroid Storm

**DOI:** 10.7759/cureus.19261

**Published:** 2021-11-04

**Authors:** Rami A Sakaan, Mary Avery Poole, Ben Long

**Affiliations:** 1 Internal Medicine, Magnolia Regional Health Center, Corinth, USA

**Keywords:** reversible, cardiogenic shock, diltiazem, atrial fibrillation, thyroid storm

## Abstract

Early recognition of underlying thyroid disease in patients presenting with new-onset tachyarrhythmia is central to management, as usual rate-control strategies can result in significant mortality and morbidity. Hyperthyroidism-induced cardiomyopathy complicated by cardiogenic shock is a life-threatening condition. Thyroid storm can lead to irreversible cardiovascular collapse and death if proper treatment is not initiated as soon as possible. In this case, we report a 44-year-old female who presented to the emergency department (ED) with the chief complaints of anxiety and palpitations. Her past medical history was significant for anxiety, but she was otherwise healthy. An electrocardiogram (ECG) in the ED demonstrated atrial fibrillation (A. fib) with rapid ventricular response (RVR) felt to be secondary to thyroid storm based on the Burch-Wartofsky point scale (BWPS), which is a quantitative diagnostic tool that uses clinical manifestation in diagnosing thyroid storm. The patient was initially managed with a continuous infusion of diltiazem to achieve rate control with a goal heart rate <115 beats per minute (bpm). Shortly after initiating the infusion, the patient developed signs and symptoms consistent with cardiogenic shock. Bedside echocardiogram revealed an estimated ejection fraction (EF) <10% with concomitant pulmonary edema. Repeat echocardiogram within 72 hours after stopping diltiazem and starting appropriate treatment for thyroid storm showed improvement of EF to 35%.

## Introduction

This case report was presented as a poster in Southern Medical Association (SMA) on May 5, 2021. New-onset arrhythmias in young patients raise concerns for secondary causes including infection, alcohol abuse, drug intoxication, medication side effects, thyrotoxicosis, and electrolyte abnormalities among others. Initial workup should include drug screen, complete blood count (CBC) with differential, comprehensive metabolic profile (CMP), and thyroid stimulating hormone (TSH) [1]. 

Thyroid storm is a rare, life-threatening condition characterized by severe clinical manifestations of thyrotoxicosis. Cardiopulmonary failure is the most common cause of death in thyroid storm [2]. Arrhythmia is commonly one of the earliest manifestations of thyrotoxicosis that causes patients to present to the emergency department (ED) [3]. Promptly obtaining a complete physical exam, basic laboratory work, and thyroid function tests are extremely important for any patient who presents with new-onset arrhythmia, as early recognition and etiology-specific intervention are associated with improved patient outcomes.

## Case presentation

The patient is a 44-year-old Caucasian female with a past medical history of anxiety who presented to the ED with a chief complaint of intermittent palpitations for the past four to six weeks. Her symptoms had acutely worsened in the 24 hours prior to presentation. Additional review of systems (ROS) was significant for four to six months of menopausal symptoms described as hot flashes, lack of menses, and vaginal dryness. She also endorsed a 10-pound unintentional weight loss over the past four weeks. She had recently been prescribed medroxyprogesterone by her primary care provider. Patient denied any history of thyroid disease, autoimmune disease, or atrial fibrillation/atrial flutter. Patient denied any drug or alcohol abuse.

**Figure 1:**
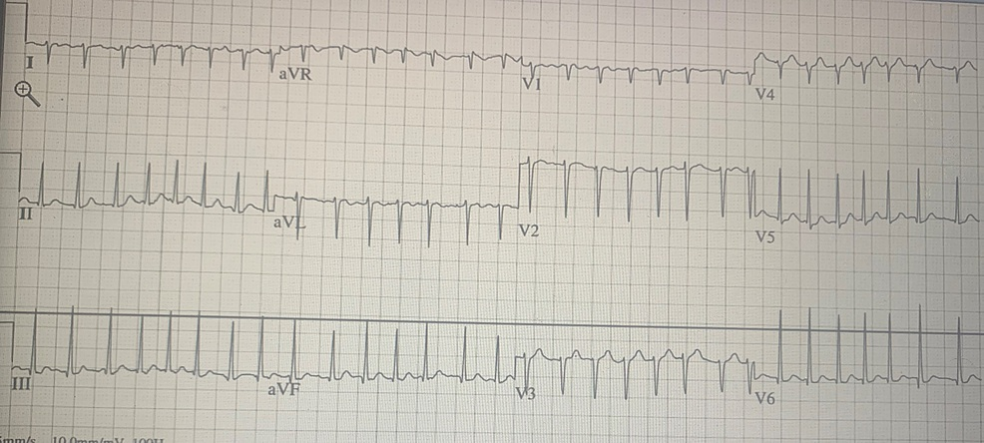
EKG showing atrial fibrillation with rapid ventricular response (RVR), with right axis deviation (RAD)

In the ED, vitals were as follows: temperature of 98.1°F (36.7°C), heart rate 185 bpm, blood pressure of 133/100 mmHg, and oxygen saturation 96% on room air. Initial CBC was significant for hemoglobin 12.7 grams per deciliter (g/dL), hematocrit 40.4%, platelet count of 143,000 cells per microliter of blood (c/μL). Prothrombin time and international normalized ratio were 13.8 seconds and 1.22, respectively. Initial CMP was not significant. Initial troponin was 0.017 nanograms per milliliter (ng/mL), urine drug test (UDS) was negative, urine pregnancy test (UPT) was negative, TSH was less than 0.02 micro-international units per milliliter (μIU/mL), free thyroxine (T4) 6.99 nanograms per deciliter of blood (ng/dL), and free triiodothyronine (T3) 22.80 picograms per milliliter of blood (pg/mL). Electrocardiogram (EKG) showed atrial fibrillation (A. fib) with rapid ventricular response (RVR) at a rate of 182 bpm as seen below in Figure 1. 

The patient was given 20 milligrams (mg) intravenous (IV) push of diltiazem, followed by continuous infusion of diltiazem. She was also started on apixaban 5 mg oral twice daily for stroke prophylaxis.

She was admitted to the intensive care unit (ICU) for closer monitoring. At the time of admission, she was hemodynamically stable and asymptomatic. Pertinent physical examination findings included a generally anxious appearing female with mildly increased work of breathing, irregularly irregular pulse, a non-tender diffusely enlarged thyroid gland without any appreciable nodularity, and mild non-pitting edema in the bilateral lower extremities.

Several hours after admission, the patient became acutely short of breath and diaphoretic. She endorsed orthopnea and dizziness, and her examination was significant for jugular venous distention and cool extremities. Vital signs at that time showed a temperature of 98.8°F (37.1°C), heart rate 114 bpm, blood pressure of 73/51 mmHg, mean arterial pressure (MAP) of 56 mmHg, respiration rate (RR) 41 b/m, and oxygen saturation 98% on room air. Diltiazem was stopped after patient’s MAP fell to less than 65 mmHg and an infusion of norepinephrine was initiated. Cardiology was consulted and dobutamine was added for ionotropic support. Transthoracic echocardiogram (TTE) revealed a severely depressed left ventricle (LV) with a calculated ejection fraction (EF) of 5-10% with global hypokinesis, with severely dilated right ventricle (RV), in addition to elevated right ventricular systolic pressure (RVSP) is consistent with mild pulmonary hypertension as seen in the four chamber view in Video 1.

**Video 1:** Four chamber view TTE revealed a severely depressed LV with an EF of 5-10% with global hypokinesis, with severely dilated RV, elevated RVSP TTE: transthoracic echocardiogram, LV: left ventricle, RV: right ventricle, EF: ejection fraction, RVSP: right ventricular systolic pressure

Endocrinology evaluated the patient at bedside and began therapy with propylthiouracil (PTU) 200 mg oral every six hours, hydrocortisone 100 mg IV three times daily, and potassium iodide/iodine (Lugol’s solution) three times daily.

The patient improved clinically within 24 hours and all vasopressors were weaned off. She was transferred from the ICU to a regular floor bed under continued observation for 48 hours. Repeat TTE at that time showed marked improvement with the left ventricular EF of 35% as seen in Video 2.

**Video 2:** TTE showing EF of 30-35% TTE: transthoracic echocardiogram, EF: ejection fraction

The patient was discharged home on methimazole 50 mg daily in addition to propranolol 40 mg four times daily with appropriate clinic follow up with endocrinology and cardiology.

## Discussion

Diagnosis of thyroid storm is based on clinical criteria alone [4]. Therefore, maintaining high clinical suspicion when laboratory evidence is suggestive of thyrotoxicosis is paramount in guiding clinical management. The hallmark symptoms of life-threatening thyroid storm include hyperpyrexia, cardiovascular dysfunction, and altered mentation. Fortunately, owing to the advancement of recognition and therapeutic strategies, the mortality rate of this disorder has dropped from nearly 100% to 10% [4]. 

**Figure 2:**
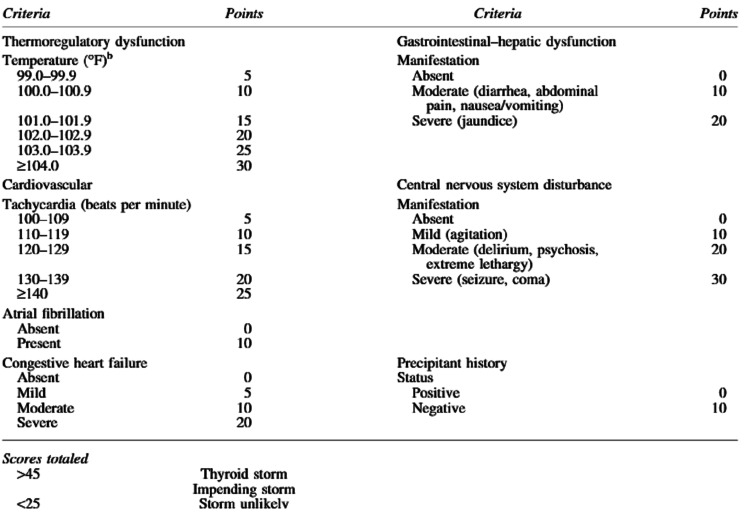
Point Scale for the Diagnosis of Thyroid Storm

Burch-Wartofsky Point Scale (BWPS) is one of the scoring systems originally developed in 1993 in an effort to delineate thyroid storm and thyrotoxicosis. This system uses clinical manifestation to guide with diagnosis. Those clinical manifestations include hyperpyrexia, tachycardia, arrhythmias, congestive heart failure, agitation, delirium, psychosis, stupor, and coma, as well as nausea, vomiting, diarrhea, hepatic failure, and the presence of an identified precipitant (Figure 2) [5].

This patient meets the criteria for thyroid storm based on the score above, as her score was around 55 points, based on heart rate greater than 140 bpm with A. fib, moderate congestive heart failure (CHF), and mild agitation. Therefore initiation of appropriate treatment for thyroid storm/thyrotoxicosis immediately after recognition of symptoms is imperative in preventing adverse outcomes. It is well known that any category of thyroid dysfunction can affect cardiac function [6].

Cardiopulmonary failure is the most common cause of death in patients presenting with thyroid storm or severe thyrotoxicosis [2]. As we see in this patient, who presented initially with A. fib with RVR, her initial EKG as shown in Figure 1 showed A. fib with RVR with right axis deviation (RAD), which is most likely due to severe pulmonary hypertension. The cause of this is secondary to thyrotoxicosis, as thyroid hormone enhance catecholamine sensitivity, causing pulmonary vasoconstriction, reduction in pulmonary vascular compliance and an increase in pulmonary vascular resistance as seen in the ECHO in Video 1 [7]. 

Given the patient had been having those symptoms intermittently for almost four to six weeks, which induced heart failure probably due to her chronic untreated hyperthyroidism. This is based on previous studies that showed heart failure in hyperthyroidism could result from thyroid hormone driven tachycardia-mediated mechanism leading to an increased level of cytosolic calcium during diastole with reduced ventricular contractility and diastolic dysfunction [8].

As hyper-adrenergic states plays a compensatory role in maintaining cardiac output and end organ perfusion in these patients, therefore atrioventricular (AV) blocker agents such as calcium-channel-blockers (CCB) and beta-blocker (BB) agents disrupt this compensatory mechanism and can cause a significant drop in cardiac output with hemodynamic instability [9].

Once diagnosed, guidelines suggest that patients with tachyarrhythmia be immediately started on BB (i.e. propranolol typically 60 to 80 mg orally every four to six hours) to maintain a heart rate below 90 bpm, in addition to thionamides such as PTU 200 mg orally every four hours or methimazole 20 mg orally every four to six hours. PTU is preferred due to its effect on decreasing conversion of T4 to T3 by blocking type 1 deiodinase, which may help in reducing serum T3 levels, as free T3 is considered the biologically active thyroid hormone that binds to thyroid hormone receptors [1]. Also, saturated solution of potassium iodide five drops orally every six hours or potassium iodide/iodine (Lugol’s solution) 10 drops every eight hours should be started one hour after starting thionamide to prevent the iodine from being used as a substrate for new hormone synthesis in patients with toxic adenoma or toxic multinodular goiter [6]. Depending on the patient’s condition and severity of the thyroid storm, administration of glucocorticoids (hydrocortisone 100 mg IV every eight hours) or cholestyramine 4 g orally four times daily can be beneficial in patients with severe presentations to reduce enterohepatic recycling of thyroid hormone [10-12].

## Conclusions

Clinicians should be cautious in prioritizing treatment in cases of new onset atrial fibrillation with rapid ventricular response and evidence of thyrotoxicosis. Since there is no universal definition to aid in diagnosing thyroid storm, high clinical suspicion must be maintained in order to initiate timely, effective treatment. Compared to previous case reports involving patients presenting with new onset A. fib secondary to underlying thyrotoxicosis/thyroid storm, the development of heart failure with reduced ejection fraction in patients with longstanding hyperthyroidism is not uncommon. Therefore, prioritizing appropriate treatment for thyroid storm rather than achieving rapid rate control may prevent AV nodal blockade induced decompensated heart failure and cardiogenic shock. 

## References

[1] Krahn AD, Klein GJ, Kerr CR (1996). How useful is thyroid function testing in patients with recent-onset atrial fibrillation? The Canadian Registry of Atrial Fibrillation Investigators. Arch Intern Med.

[2] Burch HB, Wartofsky L (1993). Life-threatening thyrotoxicosis. Thyroid storm. Endocrinol Metab Clin North Am.

[3] Martinez-Diaz GJ, Formaker C, Hsia R (2012). Atrial fibrillation from thyroid storm. J Emerg Med.

[4] Chiha M, Samarasinghe S, Kabaker AS (2015). Thyroid storm: an updated review. J Intensive Care Med.

[5] Ross DS, Burch HB, Cooper DS (2016). 2016 American Thyroid Association guidelines for diagnosis and management of hyperthyroidism and other causes of thyrotoxicosis. Thyroid.

[6] Nai Q, Ansari M, Pak S (2018). Cardiorespiratory failure in thyroid storm: case report and literature review. J Clin Med Res.

[7] Marvisi M, Balzarini L, Mancini C, Mouzakiti P (2013). Thyroid gland and pulmonary hypertension. What's the link?. Panminerva Med.

[8] Dahl P, Danzi S, Klein I (2008). Thyrotoxic cardiac disease. Curr Heart Fail Rep.

[9] Zacharia J, May T (2021). The perils of beta-blockade and the promise of venoarterial extracorporeal membrane oxygenation in managing low-output heart failure in thyroid storm: a case report. J Emerg Crit Care Med.

[10] Subahi A, Ibrahim W, Abugroun A (2018). Diltiazem-associated cardiogenic shock in thyrotoxic crisis. Am J Ther.

[11] Angell TE, Lechner MG, Nguyen CT, Salvato VL, Nicoloff JT, LoPresti JS (2015). Clinical features and hospital outcomes in thyroid storm: a retrospective cohort study. J Clin Endocrinol Metab.

[12] Abuid J, Larsen PR (1974). Triiodothyronine and thyroxine in hyperthyroidism. Comparison of the acute changes during therapy with antithyroid agents. J Clin Invest.

